# 4-[(4-Methyl­benzene­sulfonamido)­meth­yl]cyclo­hexa­necarb­oxy­lic acid

**DOI:** 10.1107/S1600536811020083

**Published:** 2011-06-04

**Authors:** Muhammad Ashfaq, Samina Iram, Mehmet Akkurt, Islam Ullah Khan, Ghulam Mustafa, Shahzad Sharif

**Affiliations:** a Department of Chemistry, University of Gujrat, H.H. Campus, Gujrat 50700, Pakistan; b Department of Physics, Faculty of Sciences, Erciyes University, 38039 Kayseri, Turkey; c Department of Chemistry, Government College University, Lahore 54000, Pakistan

## Abstract

The title compound, C_15_H_21_NO_4_S, crystallized with two independent mol­ecules in the asymmetric unit in which the dihedral angles between the mean planes of the benzene and cyclo­hexane rings are 78.3 (2) and 67.6 (2)°. The substituents of the cyclo­hexyl rings are in equatorial orientations. In the crystal, both mol­ecules form *R*_2_^2^(6) carb­oxy­lic acid inversion dimers *via* pairs of O—H⋯O hydrogen bonds. Further N—H⋯O and C—H⋯O inter­actions generate a three-dimensional network.

## Related literature

For background to tranexamic acid, see: Boylan *et al.* (1996 ▶); Nilsson (1980 ▶); Khan *et al.* (2002 ▶); Shah *et al.* (2010 ▶); Shahzadi *et al.* (2007 ▶); Svahn *et al.* (1986 ▶); Vávrová *et al.* (2005 ▶).
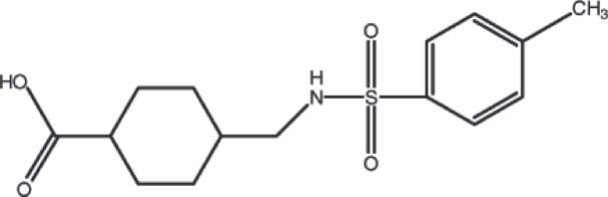


## Experimental

### 

#### Crystal data

C_15_H_21_NO_4_S
*M* *_r_* = 311.40
Triclinic, 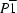

*a* = 6.0655 (5) Å
*b* = 10.3999 (9) Å
*c* = 26.468 (2) Å
α = 98.947 (3)°
β = 90.001 (4)°
γ = 104.880 (3)°
*V* = 1592.6 (2) Å^3^
*Z* = 4
Mo *K*α radiation
μ = 0.22 mm^−1^
*T* = 296 K
0.24 × 0.18 × 0.07 mm

#### Data collection

Bruker APEXII CCD diffractometer
18232 measured reflections
7641 independent reflections
3536 reflections with *I* > 2σ(*I*)
*R*_int_ = 0.059

#### Refinement

*R*[*F* ^2^ > 2σ(*F* ^2^)] = 0.076
*wR*(*F* ^2^) = 0.227
*S* = 1.02
7641 reflections
394 parameters
5 restraints
H atoms treated by a mixture of independent and constrained refinement
Δρ_max_ = 0.53 e Å^−3^
Δρ_min_ = −0.35 e Å^−3^

### 

Data collection: *APEX2* (Bruker, 2007 ▶); cell refinement: *SAINT* (Bruker, 2007 ▶); data reduction: *SAINT*; program(s) used to solve structure: *SHELXS97* (Sheldrick, 2008 ▶); program(s) used to refine structure: *SHELXL97* (Sheldrick, 2008 ▶); molecular graphics: *PLATON* (Spek, 2009 ▶); software used to prepare material for publication: *WinGX* (Farrugia, 1999 ▶) and *PLATON*.

## Supplementary Material

Crystal structure: contains datablock(s) global, I. DOI: 10.1107/S1600536811020083/hb5893sup1.cif

Structure factors: contains datablock(s) I. DOI: 10.1107/S1600536811020083/hb5893Isup2.hkl

Supplementary material file. DOI: 10.1107/S1600536811020083/hb5893Isup3.cml

Additional supplementary materials:  crystallographic information; 3D view; checkCIF report

## Figures and Tables

**Table 1:** Hydrogen-bond geometry (Å, °)

*D*—H⋯*A*	*D*—H	H⋯*A*	*D*⋯*A*	*D*—H⋯*A*
O4—H4*O*⋯O3^i^	0.84 (4)	1.81 (4)	2.638 (6)	171 (4)
O7—H7*O*⋯O8^ii^	0.81 (4)	1.87 (4)	2.664 (5)	166 (5)
N1—H1*N*⋯O6^iii^	0.86 (3)	2.15 (3)	3.001 (4)	171 (4)
N2—H2*N*⋯O2	0.84 (3)	2.09 (4)	2.924 (4)	171 (4)
C10—H10*B*⋯O6^iii^	0.97	2.57	3.446 (6)	151
C19—H19⋯O2	0.93	2.59	3.490 (5)	164

Symmetry codes: (i) 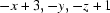; (ii) 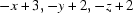; (iii) 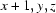.

**Fig. 1.**
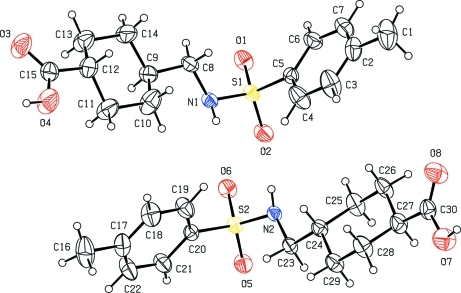
View of the two independent molecules in the asymmetric unit with displacement ellipsoids for non-H atoms drawn at the 30% probability level.

**Fig. 2.**
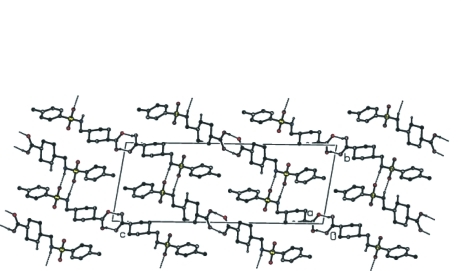
The packing and hydrogen bonding of (I), viewed down a axis. Hydrogen atoms not involved in hydrogen bonding have been omitted for clarity.

## supplementary crystallographic information

### Comment

Tranexamic acid, is a synthetic amino acid that is used commonly for curing abnormal bleeding in a variety of diseases (Boylan *et al.*, 1996; Nilsson, 1980). Number of Scientists derivatized this drug, evaluated their activities and found most of them superior to the parent drug (Svahn *et al.*, 1986; Khan *et al.*, 2002; Vávrová *et al.*, 2005; Shahzadi *et al.*, 2007; Shah *et al.*, 2010).

As seen in Fig. 1, the title compound (I) crystalizes in the triclinic space group P-1 with two independent molecules in the asymmetric unit. The dihedral angle is 64.7 (2) ° between the benzene rings (C2–C7 and C17–C22) of two independent molecules in the asymmetric unit. The cyclohexane rings (C9–C14 and C24–C29) of two independent molecules have a chair configuration [the puckering parameters are Q_T_ = 0.561 (5) Å, θ = 174.4 (5) °, φ = 353 (6) ° and Q_T_ = 0.563 (5) Å, θ = 0.8 (5) °, φ = 139 (29) °, respectively].

In the crystal structure, intermolecular N—H···O, O—H···O and C—H···O interactions help to stabilize the crystal structure (Table 1, Fig. 2).

### Experimental

4-toluenesulfonyl chloride (1.21 g, 6.36 mmol) was added to the solution of tranexamic acid (1.0 g, 6.36 mmol) in distilled water (10 ml). The reaction mixture was stirred at room temperature at pH 8–9, which was adjusted by 1 *M* sodium carbonate solution. After completion of the reaction which was observed by the consumption of 4-toluenesulfonyl chloride, the pH was adjusted at 2–3 using 1 N HCl solution, which results the formation of precipiates, which were filtered off and dried. The product was recrystallized using methanol to yield colourless plates of (I).

### Refinement

The N and O-bound H atoms were located in a difference Fourier map and their positional parameters were restrained [N1—H1N = 0.86 (3), N2—H2N = 0.84 (3), O4—H4O = 0.84 (4) and O7—H7O = 0.81 (4) Å]. Their displacement parameters were refined using a riding model, with *U*_iso_(H) = 1.2*U*_eq_(N) or 1.5*U*_eq_(O). The remaining H atoms were placed at calculated positions and allowed to ride on their carrier atoms with C—H = 0.93 - 0.98 Å, and *U*_iso_ = 1.2*U*_eq_(C) for CH and CH_2_ groups and *U*_iso_ = 1.5*U*_eq_(C) for CH_3_ group.

### Crystal data

**Table:**

C_15_H_21_NO_4_S	*Z* = 4
*M**_r_* = 311.40	*F*(000) = 664
Triclinic, *P*1	*D*_x_ = 1.299 Mg m^−^^3^
Hall symbol: -P 1	Mo *K*α radiation, λ = 0.71073 Å
*a* = 6.0655 (5) Å	Cell parameters from 2057 reflections
*b* = 10.3999 (9) Å	θ = 2.8–21.5°
*c* = 26.468 (2) Å	µ = 0.22 mm^−^^1^
α = 98.947 (3)°	*T* = 296 K
β = 90.001 (4)°	Plate, colourless
γ = 104.880 (3)°	0.24 × 0.18 × 0.07 mm
*V* = 1592.6 (2) Å^3^	

### Data collection

**Table:**

Bruker APEXII CCD diffractometer	3536 reflections with *I* > 2σ(*I*)
Radiation source: sealed tube	*R*_int_ = 0.059
graphite	θ_max_ = 28.3°, θ_min_ = 2.3°
φ and ω scans	*h* = −5→8
18232 measured reflections	*k* = −13→13
7641 independent reflections	*l* = −33→35

### Refinement

**Table:**

Refinement on *F*^2^	Primary atom site location: structure-invariant direct methods
Least-squares matrix: full	Secondary atom site location: difference Fourier map
*R*[*F*^2^ > 2σ(*F*^2^)] = 0.076	Hydrogen site location: inferred from neighbouring sites
*wR*(*F*^2^) = 0.227	H atoms treated by a mixture of independent and constrained refinement
*S* = 1.02	*w* = 1/[σ^2^(*F*_o_^2^) + (0.1014*P*)^2^] where *P* = (*F*_o_^2^ + 2*F*_c_^2^)/3
7641 reflections	(Δ/σ)_max_ = 0.001
394 parameters	Δρ_max_ = 0.53 e Å^−^^3^
5 restraints	Δρ_min_ = −0.35 e Å^−^^3^

### Special details

**Table:**

Geometry. Bond distances, angles *etc*. have been calculated using the rounded fractional coordinates. All su's are estimated from the variances of the (full) variance-covariance matrix. The cell e.s.d.'s are taken into account in the estimation of distances, angles and torsion angles
Refinement. Refinement on *F*^2^ for ALL reflections except those flagged by the user for potential systematic errors. Weighted *R*-factors *wR* and all goodnesses of fit *S* are based on *F*^2^, conventional *R*-factors *R* are based on *F*, with *F* set to zero for negative *F*^2^. The observed criterion of *F*^2^ > σ(*F*^2^) is used only for calculating -*R*-factor-obs *etc*. and is not relevant to the choice of reflections for refinement. *R*-factors based on *F*^2^ are statistically about twice as large as those based on *F*, and *R*-factors based on ALL data will be even larger.

### Fractional atomic coordinates and isotropic or equivalent isotropic displacement parameters (Å^2^)

**Table:**

	*x*	*y*	*z*	*U*_iso_*/*U*_eq_	
S1	0.59294 (16)	0.36920 (10)	0.78560 (4)	0.0462 (3)	
O1	0.4029 (4)	0.2571 (3)	0.78800 (10)	0.0609 (10)	
O2	0.5563 (5)	0.4991 (3)	0.78533 (10)	0.0647 (11)	
O3	1.2671 (7)	−0.0636 (4)	0.53183 (13)	0.1013 (17)	
O4	1.4863 (7)	0.1415 (4)	0.54321 (14)	0.1083 (17)	
N1	0.7227 (5)	0.3365 (3)	0.73371 (11)	0.0450 (11)	
C1	1.2352 (11)	0.4191 (6)	0.96605 (19)	0.114 (3)	
C2	1.0735 (9)	0.4061 (5)	0.92107 (17)	0.0733 (19)	
C3	1.1413 (8)	0.4766 (6)	0.88168 (19)	0.093 (2)	
C4	0.9963 (8)	0.4656 (5)	0.84050 (17)	0.0814 (19)	
C5	0.7805 (6)	0.3824 (4)	0.83802 (13)	0.0442 (12)	
C6	0.7115 (8)	0.3108 (4)	0.87682 (15)	0.0656 (17)	
C7	0.8597 (10)	0.3241 (5)	0.91804 (17)	0.082 (2)	
C8	0.7808 (7)	0.2069 (4)	0.72344 (14)	0.0562 (16)	
C9	0.8747 (8)	0.1807 (4)	0.67113 (16)	0.0592 (17)	
C10	1.0961 (9)	0.2721 (5)	0.6634 (2)	0.092 (2)	
C11	1.1912 (10)	0.2368 (4)	0.61059 (19)	0.093 (2)	
C12	1.2054 (9)	0.0906 (4)	0.60319 (17)	0.0717 (19)	
C13	0.9806 (9)	−0.0001 (5)	0.60706 (19)	0.084 (2)	
C14	0.8840 (10)	0.0355 (4)	0.65955 (18)	0.086 (2)	
C15	1.3243 (9)	0.0523 (5)	0.55579 (17)	0.0653 (17)	
S2	0.25305 (15)	0.71011 (9)	0.71862 (3)	0.0417 (3)	
O5	0.1844 (5)	0.8290 (3)	0.71441 (10)	0.0600 (10)	
O6	0.0813 (4)	0.5889 (3)	0.72280 (9)	0.0562 (9)	
O7	1.3989 (6)	1.0912 (4)	0.95942 (13)	0.0918 (17)	
O8	1.2587 (6)	0.8845 (3)	0.97377 (12)	0.0874 (12)	
N2	0.4188 (5)	0.7396 (3)	0.76897 (10)	0.0416 (10)	
C16	0.7982 (10)	0.5973 (6)	0.53281 (18)	0.110 (3)	
C17	0.6599 (8)	0.6246 (5)	0.57895 (16)	0.0673 (17)	
C18	0.6913 (8)	0.5789 (5)	0.62363 (18)	0.0771 (19)	
C19	0.5698 (8)	0.6030 (5)	0.66593 (16)	0.0675 (17)	
C20	0.4129 (6)	0.6759 (4)	0.66451 (13)	0.0429 (11)	
C21	0.3779 (8)	0.7221 (4)	0.61988 (15)	0.0649 (17)	
C22	0.4988 (10)	0.6941 (5)	0.57743 (17)	0.084 (2)	
C23	0.6137 (6)	0.8615 (4)	0.77586 (13)	0.0464 (12)	
C24	0.7819 (6)	0.8593 (4)	0.81700 (13)	0.0487 (12)	
C25	0.6862 (7)	0.8384 (5)	0.86771 (15)	0.0705 (18)	
C26	0.8735 (7)	0.8386 (5)	0.90713 (15)	0.0674 (16)	
C27	1.0635 (7)	0.9679 (4)	0.91159 (15)	0.0641 (17)	
C28	1.1607 (7)	0.9884 (5)	0.86120 (15)	0.0728 (19)	
C29	0.9747 (7)	0.9873 (4)	0.82208 (14)	0.0597 (14)	
C30	1.2480 (7)	0.9766 (5)	0.95100 (15)	0.0604 (16)	
H1A	1.27680	0.51080	0.98320	0.1720*	
H1B	1.36980	0.39360	0.95410	0.1720*	
H1C	1.16170	0.36110	0.98940	0.1720*	
H1N	0.831 (6)	0.408 (3)	0.7338 (16)	0.0790*	
H3	1.28830	0.53290	0.88290	0.1110*	
H4	1.04500	0.51480	0.81430	0.0980*	
H4O	1.555 (6)	0.119 (3)	0.5172 (13)	0.0980*	
H6	0.56550	0.25330	0.87550	0.0790*	
H7	0.81110	0.27530	0.94440	0.0990*	
H8A	0.64520	0.13520	0.72630	0.0670*	
H8B	0.89300	0.20490	0.74920	0.0670*	
H9	0.76490	0.19270	0.64630	0.0710*	
H10A	1.20500	0.26930	0.68980	0.1100*	
H10B	1.07990	0.36340	0.66720	0.1100*	
H11A	1.09210	0.24870	0.58390	0.1120*	
H11B	1.34170	0.29630	0.60840	0.1120*	
H12	1.29950	0.08310	0.63210	0.0860*	
H13A	0.99250	−0.09240	0.60260	0.1010*	
H13B	0.87800	0.00720	0.58020	0.1010*	
H14A	0.73120	−0.02250	0.66060	0.1030*	
H14B	0.97800	0.01770	0.68590	0.1030*	
H2N	0.442 (7)	0.666 (3)	0.7742 (15)	0.0720*	
H7O	1.502 (6)	1.085 (5)	0.9778 (16)	0.0900*	
H16A	0.71020	0.59260	0.50210	0.1650*	
H16B	0.83640	0.51320	0.53270	0.1650*	
H16C	0.93570	0.66870	0.53430	0.1650*	
H18	0.79890	0.52980	0.62520	0.0930*	
H19	0.59380	0.56980	0.69550	0.0810*	
H21	0.27220	0.77250	0.61850	0.0780*	
H22	0.46980	0.72310	0.54720	0.1010*	
H23A	0.55680	0.94080	0.78460	0.0560*	
H23B	0.69050	0.86770	0.74380	0.0560*	
H24	0.84850	0.78400	0.80520	0.0580*	
H25A	0.57100	0.75300	0.86400	0.0840*	
H25B	0.61330	0.90940	0.88010	0.0840*	
H26A	0.80760	0.83020	0.94020	0.0810*	
H26B	0.93570	0.76190	0.89670	0.0810*	
H27	0.99440	1.04250	0.92290	0.0770*	
H28A	1.23410	0.91740	0.84910	0.0880*	
H28B	1.27560	1.07390	0.86490	0.0880*	
H29A	0.91360	1.06450	0.83240	0.0720*	
H29B	1.04130	0.99550	0.78900	0.0720*	

### Atomic displacement parameters (Å^2^)

**Table:**

	*U*^11^	*U*^22^	*U*^33^	*U*^12^	*U*^13^	*U*^23^
S1	0.0430 (5)	0.0572 (6)	0.0446 (6)	0.0194 (5)	0.0095 (4)	0.0158 (4)
O1	0.0422 (15)	0.080 (2)	0.0572 (17)	0.0050 (14)	0.0078 (13)	0.0194 (14)
O2	0.079 (2)	0.0739 (19)	0.0602 (18)	0.0466 (17)	0.0198 (15)	0.0231 (15)
O3	0.116 (3)	0.082 (3)	0.093 (3)	0.018 (2)	0.046 (2)	−0.012 (2)
O4	0.123 (3)	0.091 (3)	0.099 (3)	0.019 (2)	0.064 (3)	−0.006 (2)
N1	0.0477 (19)	0.051 (2)	0.0425 (17)	0.0195 (15)	0.0098 (15)	0.0149 (15)
C1	0.113 (5)	0.165 (6)	0.072 (4)	0.063 (4)	−0.031 (3)	−0.006 (3)
C2	0.074 (3)	0.095 (4)	0.054 (3)	0.039 (3)	−0.010 (2)	−0.007 (3)
C3	0.051 (3)	0.151 (5)	0.067 (3)	0.010 (3)	−0.007 (2)	0.018 (3)
C4	0.053 (3)	0.123 (4)	0.064 (3)	0.002 (3)	0.004 (2)	0.038 (3)
C5	0.045 (2)	0.049 (2)	0.041 (2)	0.0157 (18)	0.0083 (17)	0.0083 (17)
C6	0.071 (3)	0.064 (3)	0.056 (3)	−0.001 (2)	0.000 (2)	0.023 (2)
C7	0.105 (4)	0.089 (4)	0.053 (3)	0.018 (3)	−0.006 (3)	0.025 (3)
C8	0.073 (3)	0.057 (3)	0.047 (2)	0.025 (2)	0.012 (2)	0.0202 (19)
C9	0.068 (3)	0.058 (3)	0.060 (3)	0.027 (2)	0.023 (2)	0.017 (2)
C10	0.071 (3)	0.086 (4)	0.105 (4)	0.014 (3)	0.026 (3)	−0.016 (3)
C11	0.111 (4)	0.061 (3)	0.098 (4)	0.014 (3)	0.064 (4)	0.004 (3)
C12	0.082 (4)	0.073 (3)	0.065 (3)	0.033 (3)	0.022 (3)	0.004 (2)
C13	0.090 (4)	0.067 (3)	0.093 (4)	0.027 (3)	0.030 (3)	−0.002 (3)
C14	0.119 (5)	0.059 (3)	0.086 (4)	0.032 (3)	0.050 (3)	0.017 (3)
C15	0.075 (3)	0.069 (3)	0.058 (3)	0.029 (3)	0.020 (3)	0.011 (3)
S2	0.0352 (5)	0.0511 (6)	0.0402 (5)	0.0129 (4)	0.0003 (4)	0.0085 (4)
O5	0.0653 (18)	0.0715 (19)	0.0554 (17)	0.0386 (15)	−0.0008 (14)	0.0119 (14)
O6	0.0385 (14)	0.0642 (18)	0.0570 (16)	−0.0050 (13)	−0.0009 (12)	0.0134 (13)
O7	0.080 (3)	0.092 (3)	0.093 (3)	0.000 (2)	−0.045 (2)	0.0209 (19)
O8	0.077 (2)	0.094 (2)	0.085 (2)	−0.0004 (19)	−0.0317 (18)	0.0339 (19)
N2	0.0432 (17)	0.0455 (19)	0.0356 (16)	0.0095 (15)	−0.0033 (13)	0.0088 (14)
C16	0.086 (4)	0.163 (6)	0.064 (3)	0.018 (4)	0.027 (3)	−0.007 (3)
C17	0.053 (3)	0.086 (3)	0.050 (3)	0.003 (2)	0.005 (2)	−0.003 (2)
C18	0.062 (3)	0.105 (4)	0.071 (3)	0.038 (3)	0.010 (3)	0.007 (3)
C19	0.072 (3)	0.094 (3)	0.053 (3)	0.046 (3)	0.017 (2)	0.021 (2)
C20	0.0376 (19)	0.048 (2)	0.040 (2)	0.0055 (17)	−0.0024 (16)	0.0070 (17)
C21	0.077 (3)	0.080 (3)	0.049 (3)	0.035 (3)	0.007 (2)	0.020 (2)
C22	0.102 (4)	0.114 (4)	0.044 (3)	0.036 (3)	0.012 (3)	0.022 (3)
C23	0.047 (2)	0.053 (2)	0.037 (2)	0.0067 (18)	−0.0012 (16)	0.0109 (17)
C24	0.043 (2)	0.056 (2)	0.045 (2)	0.0076 (18)	−0.0065 (17)	0.0111 (18)
C25	0.047 (2)	0.103 (4)	0.054 (3)	0.003 (2)	0.000 (2)	0.018 (2)
C26	0.054 (3)	0.094 (3)	0.046 (2)	−0.004 (2)	−0.009 (2)	0.026 (2)
C27	0.057 (3)	0.076 (3)	0.051 (3)	0.003 (2)	−0.015 (2)	0.009 (2)
C28	0.052 (3)	0.098 (4)	0.057 (3)	−0.007 (2)	−0.003 (2)	0.023 (2)
C29	0.048 (2)	0.072 (3)	0.052 (2)	−0.003 (2)	−0.0085 (19)	0.020 (2)
C30	0.054 (3)	0.073 (3)	0.046 (2)	0.005 (2)	−0.005 (2)	0.004 (2)

### Geometric parameters (Å, °)

**Table:**

S1—O1	1.423 (3)	C10—H10B	0.9700
S1—O2	1.425 (3)	C10—H10A	0.9700
S1—N1	1.617 (3)	C11—H11A	0.9700
S1—C5	1.760 (4)	C11—H11B	0.9700
S2—N2	1.611 (3)	C12—H12	0.9800
S2—O5	1.422 (3)	C13—H13A	0.9700
S2—O6	1.434 (3)	C13—H13B	0.9700
S2—C20	1.771 (4)	C14—H14B	0.9700
O3—C15	1.234 (6)	C14—H14A	0.9700
O4—C15	1.251 (7)	C16—C17	1.513 (7)
O4—H4O	0.84 (4)	C17—C18	1.372 (7)
O7—C30	1.290 (6)	C17—C22	1.360 (8)
O8—C30	1.223 (6)	C18—C19	1.366 (7)
O7—H7O	0.81 (4)	C19—C20	1.364 (6)
N1—C8	1.465 (5)	C20—C21	1.379 (5)
N1—H1N	0.86 (3)	C21—C22	1.376 (7)
N2—C23	1.482 (5)	C23—C24	1.499 (5)
N2—H2N	0.84 (3)	C24—C29	1.517 (6)
C1—C2	1.510 (8)	C24—C25	1.487 (5)
C2—C3	1.370 (7)	C25—C26	1.542 (6)
C2—C7	1.351 (8)	C26—C27	1.518 (6)
C3—C4	1.373 (7)	C27—C28	1.482 (6)
C4—C5	1.366 (6)	C27—C30	1.506 (6)
C5—C6	1.365 (5)	C28—C29	1.528 (6)
C6—C7	1.381 (7)	C16—H16A	0.9600
C8—C9	1.509 (6)	C16—H16B	0.9600
C9—C14	1.509 (6)	C16—H16C	0.9600
C9—C10	1.468 (7)	C18—H18	0.9300
C10—C11	1.540 (7)	C19—H19	0.9300
C11—C12	1.527 (6)	C21—H21	0.9300
C12—C15	1.498 (7)	C22—H22	0.9300
C12—C13	1.459 (7)	C23—H23A	0.9700
C13—C14	1.537 (7)	C23—H23B	0.9700
C1—H1A	0.9600	C24—H24	0.9800
C1—H1B	0.9600	C25—H25A	0.9700
C1—H1C	0.9600	C25—H25B	0.9700
C3—H3	0.9300	C26—H26A	0.9700
C4—H4	0.9300	C26—H26B	0.9700
C6—H6	0.9300	C27—H27	0.9800
C7—H7	0.9300	C28—H28A	0.9700
C8—H8B	0.9700	C28—H28B	0.9700
C8—H8A	0.9700	C29—H29A	0.9700
C9—H9	0.9800	C29—H29B	0.9700
			
O1—S1—O2	119.56 (18)	C14—C13—H13A	110.00
O1—S1—N1	107.94 (16)	C12—C13—H13A	110.00
O1—S1—C5	107.47 (18)	C12—C13—H13B	110.00
O2—S1—N1	105.84 (17)	C14—C13—H13B	110.00
O2—S1—C5	107.52 (18)	H13A—C13—H13B	108.00
N1—S1—C5	108.07 (17)	C13—C14—H14B	109.00
N2—S2—C20	108.08 (17)	H14A—C14—H14B	108.00
O5—S2—N2	108.46 (16)	C9—C14—H14B	109.00
O5—S2—C20	107.58 (18)	C13—C14—H14A	109.00
O5—S2—O6	118.87 (18)	C9—C14—H14A	109.00
O6—S2—C20	107.80 (17)	C16—C17—C18	121.1 (5)
O6—S2—N2	105.65 (15)	C16—C17—C22	121.2 (4)
C15—O4—H4O	117 (2)	C18—C17—C22	117.7 (4)
C30—O7—H7O	109 (4)	C17—C18—C19	122.2 (5)
S1—N1—C8	118.0 (2)	C18—C19—C20	119.7 (4)
S1—N1—H1N	104 (3)	S2—C20—C19	121.1 (3)
C8—N1—H1N	118 (2)	S2—C20—C21	119.9 (3)
S2—N2—C23	118.2 (2)	C19—C20—C21	119.0 (4)
S2—N2—H2N	108 (3)	C20—C21—C22	120.3 (4)
C23—N2—H2N	119 (3)	C17—C22—C21	121.1 (4)
C3—C2—C7	118.0 (5)	N2—C23—C24	112.1 (3)
C1—C2—C3	120.6 (5)	C25—C24—C29	110.7 (3)
C1—C2—C7	121.4 (5)	C23—C24—C29	109.0 (3)
C2—C3—C4	121.2 (5)	C23—C24—C25	115.8 (3)
C3—C4—C5	120.1 (4)	C24—C25—C26	111.5 (4)
S1—C5—C6	120.5 (3)	C25—C26—C27	110.6 (4)
C4—C5—C6	119.3 (4)	C26—C27—C28	111.3 (3)
S1—C5—C4	120.3 (3)	C26—C27—C30	113.2 (4)
C5—C6—C7	119.7 (4)	C28—C27—C30	111.1 (4)
C2—C7—C6	121.8 (4)	C27—C28—C29	110.9 (4)
N1—C8—C9	113.0 (3)	C24—C29—C28	112.0 (3)
C10—C9—C14	111.0 (4)	O7—C30—O8	121.4 (4)
C8—C9—C14	109.4 (4)	O7—C30—C27	114.5 (4)
C8—C9—C10	115.1 (4)	O8—C30—C27	124.2 (4)
C9—C10—C11	113.3 (4)	C17—C16—H16A	110.00
C10—C11—C12	109.2 (4)	C17—C16—H16B	110.00
C13—C12—C15	113.7 (4)	C17—C16—H16C	109.00
C11—C12—C15	113.0 (4)	H16A—C16—H16B	109.00
C11—C12—C13	110.5 (5)	H16A—C16—H16C	109.00
C12—C13—C14	110.3 (4)	H16B—C16—H16C	109.00
C9—C14—C13	113.0 (4)	C17—C18—H18	119.00
O4—C15—C12	116.8 (4)	C19—C18—H18	119.00
O3—C15—O4	122.6 (5)	C18—C19—H19	120.00
O3—C15—C12	120.5 (5)	C20—C19—H19	120.00
C2—C1—H1C	109.00	C20—C21—H21	120.00
H1A—C1—H1B	110.00	C22—C21—H21	120.00
H1A—C1—H1C	110.00	C17—C22—H22	119.00
H1B—C1—H1C	109.00	C21—C22—H22	119.00
C2—C1—H1B	109.00	N2—C23—H23A	109.00
C2—C1—H1A	109.00	N2—C23—H23B	109.00
C4—C3—H3	119.00	C24—C23—H23A	109.00
C2—C3—H3	119.00	C24—C23—H23B	109.00
C3—C4—H4	120.00	H23A—C23—H23B	108.00
C5—C4—H4	120.00	C23—C24—H24	107.00
C7—C6—H6	120.00	C25—C24—H24	107.00
C5—C6—H6	120.00	C29—C24—H24	107.00
C2—C7—H7	119.00	C24—C25—H25A	109.00
C6—C7—H7	119.00	C24—C25—H25B	109.00
H8A—C8—H8B	108.00	C26—C25—H25A	109.00
N1—C8—H8B	109.00	C26—C25—H25B	109.00
C9—C8—H8B	109.00	H25A—C25—H25B	108.00
N1—C8—H8A	109.00	C25—C26—H26A	109.00
C9—C8—H8A	109.00	C25—C26—H26B	110.00
C10—C9—H9	107.00	C27—C26—H26A	110.00
C14—C9—H9	107.00	C27—C26—H26B	110.00
C8—C9—H9	107.00	H26A—C26—H26B	108.00
C9—C10—H10A	109.00	C26—C27—H27	107.00
C9—C10—H10B	109.00	C28—C27—H27	107.00
C11—C10—H10A	109.00	C30—C27—H27	107.00
C11—C10—H10B	109.00	C27—C28—H28A	109.00
H10A—C10—H10B	108.00	C27—C28—H28B	109.00
C10—C11—H11B	110.00	C29—C28—H28A	109.00
C12—C11—H11B	110.00	C29—C28—H28B	110.00
C12—C11—H11A	110.00	H28A—C28—H28B	108.00
C10—C11—H11A	110.00	C24—C29—H29A	109.00
H11A—C11—H11B	108.00	C24—C29—H29B	109.00
C13—C12—H12	106.00	C28—C29—H29A	109.00
C15—C12—H12	106.00	C28—C29—H29B	109.00
C11—C12—H12	106.00	H29A—C29—H29B	108.00
			
O1—S1—N1—C8	−50.3 (3)	C9—C10—C11—C12	−55.7 (6)
O2—S1—N1—C8	−179.4 (3)	C10—C11—C12—C13	58.8 (5)
C5—S1—N1—C8	65.7 (3)	C10—C11—C12—C15	−172.5 (4)
O1—S1—C5—C4	168.2 (3)	C11—C12—C15—O3	−147.3 (5)
O1—S1—C5—C6	−12.5 (4)	C11—C12—C15—O4	35.0 (7)
O2—S1—C5—C4	−61.9 (4)	C13—C12—C15—O4	162.0 (5)
O2—S1—C5—C6	117.5 (4)	C15—C12—C13—C14	172.9 (4)
N1—S1—C5—C4	51.9 (4)	C13—C12—C15—O3	−20.3 (7)
N1—S1—C5—C6	−128.7 (3)	C11—C12—C13—C14	−58.8 (5)
O5—S2—C20—C21	−25.3 (4)	C12—C13—C14—C9	55.4 (6)
O6—S2—C20—C19	−75.4 (4)	C16—C17—C18—C19	179.5 (5)
O6—S2—C20—C21	104.0 (4)	C22—C17—C18—C19	−1.1 (8)
N2—S2—C20—C19	38.4 (4)	C16—C17—C22—C21	−178.1 (5)
N2—S2—C20—C21	−142.3 (3)	C18—C17—C22—C21	2.5 (8)
O5—S2—N2—C23	−50.6 (3)	C17—C18—C19—C20	−0.7 (8)
O6—S2—N2—C23	−179.1 (3)	C18—C19—C20—S2	−179.7 (4)
C20—S2—N2—C23	65.7 (3)	C18—C19—C20—C21	1.0 (7)
O5—S2—C20—C19	155.3 (4)	S2—C20—C21—C22	−179.0 (4)
S1—N1—C8—C9	172.9 (3)	C19—C20—C21—C22	0.4 (7)
S2—N2—C23—C24	−166.7 (2)	C20—C21—C22—C17	−2.2 (7)
C1—C2—C3—C4	179.9 (5)	N2—C23—C24—C25	−54.0 (5)
C7—C2—C3—C4	0.6 (8)	N2—C23—C24—C29	−179.6 (3)
C3—C2—C7—C6	−0.2 (8)	C23—C24—C25—C26	−179.7 (4)
C1—C2—C7—C6	−179.4 (5)	C29—C24—C25—C26	−55.0 (5)
C2—C3—C4—C5	−0.5 (8)	C23—C24—C29—C28	−176.6 (3)
C3—C4—C5—S1	179.4 (4)	C25—C24—C29—C28	55.0 (4)
C3—C4—C5—C6	0.0 (7)	C24—C25—C26—C27	55.8 (5)
S1—C5—C6—C7	−178.9 (4)	C25—C26—C27—C28	−56.1 (5)
C4—C5—C6—C7	0.4 (7)	C25—C26—C27—C30	177.9 (3)
C5—C6—C7—C2	−0.4 (8)	C26—C27—C28—C29	56.0 (5)
N1—C8—C9—C10	65.8 (5)	C30—C27—C28—C29	−176.9 (4)
N1—C8—C9—C14	−168.5 (4)	C26—C27—C30—O7	−173.0 (4)
C14—C9—C10—C11	52.0 (5)	C26—C27—C30—O8	7.0 (6)
C8—C9—C14—C13	−179.3 (4)	C28—C27—C30—O7	60.9 (5)
C8—C9—C10—C11	176.9 (4)	C28—C27—C30—O8	−119.1 (5)
C10—C9—C14—C13	−51.3 (6)	C27—C28—C29—C24	−55.5 (5)

### Hydrogen-bond geometry (Å, °)

**Table:**

*D*—H···*A*	*D*—H	H···*A*	*D*···*A*	*D*—H···*A*
O4—H4O···O3^i^	0.84 (4)	1.81 (4)	2.638 (6)	171 (4)
O7—H7O···O8^ii^	0.81 (4)	1.87 (4)	2.664 (5)	166 (5)
N1—H1N···O6^iii^	0.86 (3)	2.15 (3)	3.001 (4)	171 (4)
N2—H2N···O2	0.84 (3)	2.09 (4)	2.924 (4)	171 (4)
C10—H10B···O6^iii^	0.97	2.57	3.446 (6)	151
C19—H19···O2	0.93	2.59	3.490 (5)	164

Symmetry codes: (i) −*x*+3, −*y*, −*z*+1; (ii) −*x*+3, −*y*+2, −*z*+2; (iii) *x*+1, *y*, *z*.
